# La escalada tecnológica de la práctica médica y los impactos en la relación del médico con su trabajo

**DOI:** 10.18294/sc.2025.5762

**Published:** 2025-07-10

**Authors:** Lilia Blima Schraiber

**Affiliations:** 1 Médica, Doctora en Medicina. Profesora Senior, Departamento de Medicina Preventiva, Faculdade de Medicina, Universidade de São Paulo. Investigadora 1A, Conselho Nacional de Desenvolvimento Científico e Tecnológico (CNPq), Brasil. malenalenta@psi.uba.ar Universidade de São Paulo Departamento de Medicina Preventiva Faculdade de Medicina Universidade de São Paulo Brazil malenalenta@psi.uba.ar; Conselho Nacional de Desenvolvimento Científico e Tecnológico (CNPq)

**Keywords:** Práctica Médica, Tecnología Biomédica, Autonomía Profesional, Salud Colectiva, Brasil, Medical Practice, Biomedical Technology, Professional Autonomy, Collective Health, Brazil

## Abstract

Este ensayo se propone reflexionar críticamente sobre los impactos de la escalada tecnológica en la práctica médica, con especial atención a las transformaciones de su práctica profesional. Desde una perspectiva histórico-social anclada en el pensamiento crítico de la salud colectiva, particularmente en la obra de Ricardo Bruno Mendes-Gonçalves, se examina la tecnología como forma material e inmaterial que reconfigura el trabajo asistencial y reproduce el orden social. A partir del análisis de estudios previos basados en relatos de vida laboral de médicos de distintas generaciones, se abordan tres ejes centrales: la construcción y el progresivo derrumbe de la autonomía médica, la crisis de los vínculos de confianza en la práctica profesional, y la transformación del juicio clínico en favor de protocolos estandarizados. Se concluye que la tecnificación creciente ha reducido la reflexividad del acto médico, transformado la consulta en una práctica despersonalizada. Frente a esta crisis, se plantea la necesidad de recuperar la dimensión ético-política de la medicina, promoviendo decisiones compartidas y espacios de reconocimiento mutuo entre profesionales y pacientes.

## Abstract (en)

This essay aims to critically reflect on the impacts of the technological escalation in medical practice, with particular attention to the transformations of professional practice. From a historical-social perspective grounded in the critical thought of collective health - particularly the work of Ricardo Bruno Mendes-Gonçalves - technology is examined as both a material and immaterial form that reconfigures healthcare work and reproduces the social order. Based on the analysis of previous studies drawing on the work-life narratives of physicians from different generations, three central axes are addressed: the construction and progressive dismantling of medical autonomy, the crisis of trust-based relationships in professional practice, and the transformation of clinical judgment in favor of standardized protocols. The essay concludes that the growing technification has reduced the reflexivity of medical action and transformed the clinical encounter into a depersonalized practice. In the face of this crisis, it argues for the need to recover the ethical-political dimension of medicine by promoting shared decision-making and spaces of mutual recognition between professionals and patients.

## PRETENSIONES Y LÍMITES DEL TEXTO

En este texto tomo como referencia la producción intelectual de Ricardo Bruno Mendes-Gonçalves^1^, inscrita en el pensamiento crítico de la salud colectiva de la década de 1970. Adopto de Mendes-Gonçalves el concepto de tecnología como aquello que corresponde tanto a los instrumentos, en cuanto tecnología material, como al saber, en cuanto tecnología inmaterial^2^^,^^3^. Se trata, entonces, de un recurso material y simbólico que construyó la práctica médica como si fuese un mundo apartado de lo social, un mundo neutro en relación con la esfera ético-política de la vida social. Criticando esta aproximación, Mendes-Gonçalves^5^^,^^6^ aborda la tecnología como forma creativa de reproducción de lo social en el interior de la práctica técnica del trabajo clínico-asistencial, enfoque que aquí comparto.

Esa construcción de un “mundo aparte” se aferra a dicha neutralidad mediante el abordaje reduccionista de los procesos de enfermar y de los cuidados, limitándolos a cuestiones biomédicas, lo que permite concebir que la práctica médica es tan solo una intervención técnico-científica. Esta forma de concebir las cuestiones del proceso salud-enfermedad-cuidados fue definida, en el pensamiento de la salud colectiva, como un proceso de medicalización de lo social.

Donnangelo^6^ señala dos vertientes de este proceso. En una perspectiva más comúnmente explorada, la medicalización se identifica con el dominio socialmente expandido de la medicina a través de sus medicamentos -actualmente denominado medicamentalización- o de sus intervenciones asistenciales, como parte de un complejo económico-político de naturaleza industrial y comercial que, al definir socialmente la medicina, lo hace valorizando exclusivamente sus productos tecnológicos. Pero esta expansión se sustenta en otra dimensión del mismo proceso: la medicalización como una determinada lectura del enfermar y del asistir/cuidar, de modo que se produzcan como respuesta -también social- a los procesos de enfermar esos mismos productos tecnológicos.

Me refiero, así, a una mirada sobre lo social que la medicina proyecta, reduciéndolo a cuestiones que solo pueden ser abordadas desde la perspectiva biomédica, valiéndose del lenguaje de las enfermedades, a través de la patologización de eventos o comportamientos. Y, en ese sentido, la medicina termina por instituirse como un instrumento de intervención sobre lo social, lo cual se ilustra claramente con la educación sanitaria formulada en las décadas de 1950 y 1960.

Las consideraciones que expondré en el presente texto parten de otra referencia: la de que la configuración de la medicina contemporánea es un producto social e histórico. Así, su saber y su práctica, que se expresan en determinadas formas de producción de la atención médica, distribuyéndose en ciertos tipos de servicios médicos y de salud, son fruto de la historia de lo social, y no una consecuencia necesaria de propiedades técnicas de la medicina. Más bien, como señala Mendes-Gonçalves, es la medicina la que se reorienta hacia lo tecnológico como una buena respuesta, en ese nivel técnico de las prácticas de salud, para articularse con el conjunto de transformaciones de la vida en sociedad. Y si de ello resulta un tecnicismo actual, esta referencia no proviene de una medicina destinada necesariamente a ser productora de tecnologías. Al contrario, convertirse en tal productora fue la respuesta histórica de una modalidad de medicina elegida por su adecuación social a la modernidad, articulándose con los requerimientos económicos, políticos e ideológicos de la vida social moderna.

Pero, en tanto instituida (socialmente), la medicina será instituyente, como afirmó Mendes-Gonçalves respecto de la forma creativa de reproducir lo social en términos de su actividad técnica. La medicina, producto histórico de lo social, será (re)productora de lo social, al asumir y elaborar, en sus propios términos (internamente a sus configuraciones y, por tanto, de un modo “médico”), cuestiones sociales. Si en Donnangelo^6^ la medicina comunitaria se revela como la lectura medicalizadora de la pobreza y de las desigualdades de carácter económico-social, hoy en día se observan diversas incursiones de la medicina en cuestiones relacionadas con el rendimiento escolar de niñas y niños y con los comportamientos de las y los jóvenes, patologizándolos.

Además, si la medicalización de lo social revistió la práctica médica de competencias que dieran respuestas asistenciales, y proporcionó una imagen de esas competencias exclusivamente como un “éxito técnico” -que no siempre se corresponde con un verdadero “éxito práctico” frente a las dificultades y desigualdades de orden social^7^-, también le correspondió a la medicalización, en tanto saber, en su uso cotidiano en los servicios asistenciales, fundar la legítima autoridad médica frente a los procesos de enfermar y a las intervenciones sobre ellos. Hegemónico históricamente, el proceso de acelerado desarrollo y uso de tecnologías materiales, como una opción posible de modernización de la práctica médica, terminó por superar la primera configuración del trabajo médico en la modernidad -la medicina liberal de los años 1890-1930 en el país- en favor de la medicina tecnológica, configuración actual y progresivamente radicalizada en su condición tecnológica.

Así, especialmente a partir de las décadas de 1980 y 1990, se comenzó a asistir a la consolidación de la medicina tecnológica y, de forma coetánea, al derrumbe de la construcción fundante de la autonomía.

Este proceso representó, entre otros impactos sociotécnicos para la práctica médica, rupturas interactivas al interior de la práctica asistencial, generando una “crisis de confianza” en la relación de los médicos con sus pacientes, en la relación con otros profesionales y entre los propios médicos, así como en la relación del médico consigo mismo, en su modo de pensar. Esta última ruptura se caracteriza por cierta abdicación reflexiva del médico en sus juicios clínicos. En otros términos, en favor de protocolos prefijados para la acción diagnóstica y terapéutica, estaría en curso un abandono del uso criterioso de la ciencia y la tecnología en la práctica médica en relación con las contingencias del caso clínico individual, perdiéndose así el filtro de una reflexión crítica por parte del médico sobre ese uso^8^. Esto se relaciona estrechamente con la crisis moderna del pensamiento^9^^,^^10^^,^^11^ y fue bien explorado en relación con el ejercicio de la clínica por Azeredo^12^.

## AUTONOMÍA Y TECNOLOGÍA EN LA PRÁCTICA MÉDICA

La autonomía y la tecnología han sido condiciones altamente valoradas por los médicos en el ejercicio de la profesión, cuya relación, en la historia de la práctica médica, llega a la contemporaneidad como una oscilación entre el negacionismo científico -en favor de la máxima libertad tecnológica para la práctica médica (como actividad del trabajo productor de la atención médica individual)- y el tecnicismo de la acción -en favor de la mínima libertad decisoria, y de la mínima responsabilidad técnica y social resultante- en la abdicación subjetiva del pensamiento, en la renuncia a la reflexión crítica sobre el uso y la pertinencia de protocolos generales de intervención frente a la situación contingente de cada caso, en el juicio clínico que es núcleo de la actividad asistencial.

En ese sentido, haré consideraciones sobre el gran valor que, a lo largo del siglo XX, los médicos atribuyeron, por un lado, a la progresiva tecnificación de sus prácticas, con el uso creciente de tecnologías materiales diagnósticas y/o terapéuticas y en configuraciones de procesos de trabajo asistencial también progresivamente tecnicistas; y, por otro lado, a la autonomía para pensar y actuar sobre el caso individual de cada paciente. Cabe recordar que esta autonomía, que se expresa en el ejercicio más libre de las decisiones clínicas, garantizó históricamente la identificación del éxito en la atención al caso con la competencia personal de cada médico en particular^13^^,^^14^^,^^15^.

Quisiera considerar los importantes conflictos que se generan en la valorización de la coexistencia de estas dos condiciones -la autonomía y la tecnología-, conflictos que derivan de elecciones históricas hechas por los propios médicos, pero que no son percibidos por ellos en sus cotidianos de trabajo, causándoles sorpresa y extrañamiento frente a situaciones vividas en el día a día de la profesión^16^^,^^17^.

Siguiendo a Donnangelo^18^ y a Donnangelo y Pereira^6^ y, más específicamente, a Mendes-Gonçalves^5^, tomo como punto de partida la concepción de que el ejercicio profesional del médico implica la práctica clínica cotidiana en las condiciones existentes de producción de los servicios asistenciales en su mercado de trabajo. En el contexto de esas condiciones, presentaré y debatiré los desafíos que rodean la relación entre autonomía profesional y tecnología.

Abordaré esta relación mediante el examen de la creación y del derrumbe de la autonomía, basándome en el hecho histórico de que fue a través de la conformación de la medicina liberal -en la que se encuentra la máxima valorización de la autonomía de los médicos como agentes de la práctica asistencial- que la práctica médica se moderniza y se constituye la profesión médica.

Es interesante observar que la medicina liberal se instaura, paradójicamente, en medio de la configuración social de la moderna producción de bienes y servicios, que no se funda en la autonomía de los agentes de las prácticas en general, sino en su opuesto: con la industrialización como referente de la socialidad moderna, ocurre históricamente la colectivización de los trabajos afines y la doble alienación de los trabajadores respecto de sus procesos de trabajo, ya sea por la alienación del sentido social del propósito de ese trabajo -con la especialización y fragmentación de la cadena productiva para alcanzar el producto final deseado-, ya sea por la alienación de la ejecución del proceso técnico de ese trabajo, a través de la pérdida de la posesión del saber-hacer necesario para la producción de los bienes y servicios socialmente deseados. Finalmente, la posesión de ese saber-hacer será, desde la perspectiva de la producción en masa de base industrial, progresivamente incorporada por las tecnociencias como conocimiento producido en experimentos de laboratorio, distante del mundo del trabajo, y será incorporada en los equipos o máquinas resultantes de ese conocimiento científico.

Al adoptar el enfoque de la autonomía a partir de la dinámica construcción-derrumbe, traída por Eric Hobsbawm^19^ para caracterizar el contexto del históricamente “breve siglo XX”, buscaré en dicha dinámica la propia relación entre autonomía y tecnología. Así, mi enfoque es de perspectiva histórica, señalando los cambios técnicos en la práctica clínica orientados por transformaciones tecnológicas más generales en el marco de cambios sociales en la producción asistencial, como referencias que nos muestran los contextos sociotécnicos del ejercicio de la profesión. En este sentido, se trata de señalar, en la esfera de la práctica técnica, el modo en que esta práctica reproduce, en condiciones técnico-científicas, el orden social del que forma parte.

Por un lado, vale destacar, como se mencionó anteriormente, que consideraré tanto la tecnología material -traducida en equipamientos, medicamentos y otros recursos utilizados como medios en la actividad del trabajo asistencial- como la tecnología inmaterial, correspondiente a un saber operatorio que delimita la configuración o arquitectura de dicha actividad, la cual, en respuesta a la reproducción del contexto social más amplio en que se inscribe, produce una organización del trabajo que demanda el uso de esos recursos, exigiendo esos medios de trabajo y no otros. Se trata aquí de la modernización de la actividad laboral para hacer que este trabajo responda a las nuevas necesidades sociales en salud, en el surgimiento de la socialidad moderna. Esta corresponde al contexto sociotécnico que posibilita la emergencia de la clínica, nuevo saber vinculado al saber-hacer de la intervención que se construye a lo largo del siglo XIX y que, en la era moderna, pasará a regir la actividad del trabajo asistencial. Y es importante recordar que, en tanto saber operatorio, la clínica pasará a regir el uso del conocimiento científico en la actividad del trabajo asistencial, adquiriendo, en la forma de su producción y de su presentación social en la modernidad, el estatuto de una ciencia particular, bien específica: la ciencia de las técnicas, tal como diversos autores entienden la tecnología^8^^,^^20^^,^^21^.

Por otro lado, enfatizo la conexión ya mencionada entre las condiciones sociales y las condiciones técnicas de la práctica médica, defendiendo la relevancia de examinar precisamente esta relación entre lo social y lo técnico-científico en términos de su consustancialidad, para visibilizar la práctica médica como una práctica al mismo tiempo técnica y social, incluso cuando es reconocida por los propios médicos, sus agentes, únicamente como una intervención de orden técnico-científico^5^^,^^6^. Señalo ya aquí una cuestión crucial advertida por Donnangelo^6^: el hecho de que los médicos, en su práctica, reproducen el orden social en el que se insertan, respondiendo a cuestiones planteadas por ese orden social, incluso si no conceptualizan dicha reproducción. Por el contrario, el entendimiento profesional -y difundido en el sentido común de la sociedad- es que el mundo médico constituiría un mundo aparte respecto de lo social, como si se tratara de una práctica que se historiza únicamente en cuanto a sus medios de intervención (como los dispositivos tecnológicos), en una historicidad comprendida como un progreso lineal y como consecuencia de una evolución considerada natural de una técnica a otra. Tal formulación se encuentra bien desarrollada en Laura Conti^22^, y la utilizo para reiterar el pensamiento del sentido común acerca de la práctica médica, sobre lo cual dice Conti: para el sentido común, si existiera alguna historicidad en el trabajo médico, esta se referiría a los medios del trabajo asistencial, pero no a sus fines, generando la comprensión de que la finalidad social del trabajo médico sería inmutable y desde siempre orientada a salvar a los enfermos de la muerte causada por sus enfermedades.

De esta concepción deriva el hecho de que los médicos evoquen frecuentemente la perennidad de su práctica (“una práctica desde siempre” o “tan antigua como la humanidad”) y la neutralidad de dicha práctica frente a lo político y lo social. Esta postura se refuerza con el asombro que estos agentes expresan cuando se dan cuenta de que sus prácticas están reproduciendo determinados intereses socioeconómicos o que están respondiendo e involucrándose con determinadas opciones políticas, como si la política -en tanto opciones históricas dentro del abanico de posibilidades para los sujetos en sociedad- no atravesara desde siempre todas las decisiones que se toman en la vida social, lo que ciertamente incluye aquellas que se toman en el interior de la medicina^3^.

Así, por esta vía de reflexión crítica al “mundo aparte” -y comprometida de forma opuesta con el conocimiento de las conexiones entre lo técnico y lo social- podremos comprender la conquista de la autonomía del médico en el ejercicio cotidiano de la profesión como una conquista tanto técnica como ético-política. También podremos comprender las tensiones que hoy se presentan sobre esa autonomía como disputas del mismo orden -técnico y ético-político- en el campo de la medicina. Y dado que la autonomía será la base de construcción de la autoridad del médico como culturalmente soberana en el campo de la salud, podremos comprender el dominio hegemónico de la medicina en dicho campo, así como la jerarquía, entonces resultante, establecida entre las distintas prácticas profesionales que hoy conforman el trabajo en salud.

Es importante aclarar que gran parte de las consideraciones aquí presentadas son fruto de una línea de investigación sobre la relación entre el médico y la medicina, que dio lugar a dos estudios secuenciales y articulados: *O médico e seu trabalho: Limites da liberdade*^23^, y *O médico e suas interações: a crise dos vínculos de confiança*^24^, reunidos posteriormente en *El médico y la medicina: autonomía y vínculos de confianza en la práctica profesional del siglo XX*^8^. Estos estudios tuvieron como base empírica la historia de vida laboral narrada por médicos, y es a partir de esa base empírica que se derivan las reflexiones que presento en este texto.

El primer estudio fue realizado entre los años 1986-1987, en el que se entrevistó a médicos graduados entre 1930 y 1955. Es decir, médicos que iniciaron su vida profesional en la primera mitad del siglo XX y que ejercían la profesión en la ciudad de São Paulo. Se trataba de médicos que actuaban en una de las cuatro grandes áreas generales de la medicina: clínica médica, cirugía, pediatría y gineco-obstetricia.

Como continuación, ya en los últimos años del siglo XX (1996-1997), realicé el segundo estudio, entrevistando a médicos graduados entre 1980 y 1985, quienes también narraron sus trayectorias laborales y ejercían la profesión en la ciudad de São Paulo, desempeñándose lo más cerca posible de las mismas cuatro áreas médicas del estudio anterior. Y dado que los cambios históricos tuvieron un impacto singular en la vida del consultorio -y por el valor simbólico que esta tiene como expresión de la autonomía del médico- fue precisamente en esa vida laboral del consultorio donde centré mis estudios.

Los desafíos allí señalados para la profesión persisten, siempre renovados hasta el día de hoy, por lo que esos estudios aún me sirven de referencia. Sin embargo, las cuestiones entonces planteadas han recibido desarrollos más recientes. Estos adoptan recortes distintos de la práctica médica: uno centrado en la dialéctica humanización-alienación en medio de la tecnología^16^; otro recorte enfocado en las relaciones entre ciencia, tecnología y trabajo^12^; otro sobre la relación entre juicio técnico-científico y sabiduría práctica en la evaluación clínica en el ámbito psiquiátrico^25^; y también sobre la crisis contemporánea de la autoridad médica y su relación con la humanización deshumanizadora en salud y con la violencia institucional^26^^,^^27^^,^^28^. También me apoyo, en este texto, en esos estudios más recientes.

El estímulo para esta línea de estudios surgió de las múltiples transformaciones de la profesión médica en las décadas de 1970 y 1980, con impactos relevantes en el ejercicio profesional, tales como: la extensiva asalariación de los médicos; la creación de empresas de asistencia médica y seguros de salud; la gran explosión de hospitales; y el aumento de los costos asistenciales en los servicios de salud. Un papel importante en este proceso -coetáneo y articulado con estos cambios- fueron los ajustes en la organización del trabajo, surgidos desde el interior de la práctica clínica, con la creación y el desarrollo de diversos recursos tecnológicos que pasaron a ser utilizados, y con el estímulo a la progresiva especialización. Estas transformaciones ya fueron, en aquel entonces, las opciones de futuro adoptadas por los médicos especialmente vinculados al “complejo médico-industrial” y respaldadas por los médicos, en general, para la producción de servicios asistenciales y la forma moderna del ejercicio profesional.

Consideraré, entonces, específicamente ese pasado, buscando las raíces históricas de la autonomía y el movimiento de su superación, impulsado por el desarrollo de una práctica progresivamente anclada en la especialización técnico-científica: la modernización se comprometió con la construcción de una sociedad basada en especialistas^3^.

Así, en primer lugar, consideraré los procesos de construcción de la autonomía, con la configuración de la medicina liberal del pequeño productor con consultorio privado. En Brasil, este proceso se desarrolla desde fines del siglo XIX hasta la primera mitad del siglo XX. A continuación, consideraré los procesos históricos del derrumbe de aquella construcción, que se inicia en la segunda mitad del mismo siglo XX y continúa en curso hasta la actualidad, con el establecimiento de otra configuración general de la medicina, a la que llamé medicina empresarial y tecnológica. O, como afirma Mendes-Gonçalves:

“‘Medicina tecnológica’ porque el volumen, la complejidad y los costos sociales de los instrumentos de trabajo fueron volviendo inviable el estilo de práctica relativamente autónomo del doctor con su maletín [...] forzando la organización de la práctica en torno a los nuevos equipamientos, en el hospital, de modo análogo a lo que ocurría en la producción industrial. [...] La importancia fundamental del instrumento de trabajo (es decir, la clínica profundamente transformada, desarrollada y fragmentada, más el hospital y los equipos materiales contenidos en él) en la caracterización de esta práctica es lo que justifica llamarla ‘medicina tecnológica’”.^4^

Este cambio reordena la relación entre el consultorio y el hospital, en tanto espacios de práctica. Pues si en la medicina liberal la atención se centraba en el consultorio y en el domicilio del paciente, siendo el hospital una extensión de esa atención^18^, en la medicina tecnológica y empresarial la atención se centra en el hospital, y el consultorio pasa a ser un lugar de tránsito… una puerta de entrada^8^. Esta nueva configuración instaura tensiones sobre aquello que había sido construido anteriormente.

## LA CONSTRUCCIÓN DE LA AUTONOMÍA

Es interesante señalar que al analizar el proceso de derrumbe del modelo liberal fue cuando la autonomía pudo ser entendida desde tres dimensiones articuladas en la formación de un cierto todo referido a la libertad de ejercer. Y esto se debió al hecho de que la desconstrucción de cada una de esas dimensiones ocurrió en períodos cronológicamente específicos y distintos entre sí. Así, lo que parecía ser un fenómeno único, un todo indivisible de características, se reveló como una composición de tres segmentos diversos.

Uno de ellos fue el de la autonomía en el mercado de trabajo, o autonomía mercantil, y fue el primer segmento en el cual los médicos pierden la libertad de negociar directamente sus servicios. Esta pérdida ocurrió progresivamente entre los años 1960 y 1980, en el acelerado proceso de asalariación del profesional médico, que se inicia en el sector público de producción asistencial y se expande al sector privado.

Otro segmento fue el de la autonomía de los médicos para organizar y gestionar su cotidiano laboral. De este modo, tenían libertad para delimitar su jornada, incluso de forma tal que permitiera valorizar el tiempo destinado al consultorio privado. Los médicos podían, allí, regular su tiempo de trabajo, ampliándolo si era necesario en su consultorio o con las visitas domiciliarias. Estas últimas fueron muy comunes en la medicina liberal. Pero también podían ampliar su jornada en atenciones de urgencia, a veces en el domicilio del paciente, otras veces en el propio domicilio del médico. En la medicina liberal, los pacientes tenían acceso a la casa del médico y podían encontrarlo en cualquier momento en que lo necesitaran.

El tiempo de la medicina se confundía, entonces, también con el tiempo de la vida privada del profesional, lo que caracterizaba una peculiar disposición a servir al paciente y a acompañar la evolución terapéutica del caso a lo largo del tiempo, lo que hoy, en la atención primaria, llamamos longitudinalidad asistencial.

Esa autonomía relativa a la jornada de trabajo fue el segundo segmento deconstruido, en la medida en que avanzaba la pérdida de la autonomía mercantil y surgía el control de la jornada por parte de un nuevo profesional que se estaba conformando: el gerente, agente especializado en administrar trabajos, inicialmente orientado hacia la atención hospitalaria.

Por último, el tercer segmento -y en el cual ya no podemos hablar de pérdida, sino de tensiones que amenazan a los médicos en su autonomía, y que se extienden hasta la actualidad- es el segmento de la intervención técnico-científica. En él se instala el control de la práctica clínica a través de auditorías de los juicios clínicos que definen las acciones diagnósticas o terapéuticas. Las tensiones en torno a la autonomía en este segmento ponen en cuestión el propio juicio clínico, ya no entendido como un dominio unilateral del médico en las decisiones tanto terapéuticas (en el sentido más restringido de los tratamientos), como asistenciales (en el sentido del cuidado, el cual excede ampliamente el dominio de tratar).

Fueron los propios médicos quienes han intentado resistir con mayor fuerza al derrumbe del segmento de la autonomía, proceso que viene ocurriendo desde fines del siglo XX. Hoy en día, es el que concentra las discusiones en la sociedad en general. No obstante, señalé tres segmentos de la autonomía porque el ejercicio de la profesión involucra necesariamente a los tres, y las bases de la creación y desarrollo de la autonomía en la medicina liberal se dieron a partir de la articulación entre ellos, formando la configuración del conjunto clínico-asistencial que reconocemos como medicina liberal.

Aunque la desagregación de ese conjunto permitiera señalar las particularidades de cada segmento en sus transformaciones específicas, el proceso histórico se caracterizó desde sus inicios por la pérdida de las bases que fundamentaban la autonomía conquistada. Dichas bases residen en relaciones interactivas que propician vínculos de confianza. La pérdida de esos vínculos generó lo que llamé, ya en el contexto pleno de la medicina tecnológica, una crisis de la propia confianza.

Identifiqué esa crisis como una ruptura de las interacciones que el médico establecía en diversos planos y que sufrieron profundas alteraciones en la medicina tecnológica: en la relación de los médicos con los pacientes; en la relación con otros profesionales de la salud; y en la relación con su saber. En cada una de ellas, la ruptura produjo distintos impactos prácticos, con significados diversos para los vínculos entre el médico y la medicina.

Voy a examinar brevemente dos de ellos: el de la relación con el paciente y el de la relación del médico con el saber.

## EL DERRUMBE DE LO CONSTRUIDO: LA RELACIÓN ENTRE EL MÉDICO Y SU PACIENTE

“*El convenio fue muy perjudicial para la actividad del médico* [...]. *Porque el paciente que te elige viene porque alguien te recomendó o porque le gustás, pero es una recomendación positiva* [...] *La persona que va a un médico vinculado a la prepaga, en teoría, está yendo a un médico del librito de la prepaga. Abrió el librito, vio que le quedaba cerca de su casa, que le resultaba más conveniente, y fue a ese. El tipo de vínculo es totalmente distinto... Muchas veces, pasa que las consultas en las prepagas no son muy satisfactorias o, a veces, sí lo son, pero la velocidad es muy rápida.* [...] *Antes, las personas elegían a los médicos mucho por una cuestión de confianza... El sistema de la confianza quedó un poco... debilitado*”. (Dr. Antonio, gastrocirujano, entrevistado en 1997)^8^

En el plano de la relación médico-paciente, la pérdida de vínculos se produjo por la progresiva descalificación del paciente como sujeto, también portador de saber y de voz en el encuentro clínico. Por lo tanto, en la construcción de esos vínculos, el paciente tenía cierta presencia en la toma de decisiones. Sin embargo, esa descalificación de su presencia fue posible en la medida en que el conocimiento científico pareció, para los médicos, ser capaz de suplir toda la información necesaria sobre esos sujetos y sobre sus procesos de enfermar. Así, hasta la primera mitad del siglo XX, la información científica disponible parecía requerir una complementación proveniente directamente del paciente, una información vinculada a su experiencia de enfermar y también a sus posibilidades concretas de tratamiento.

Obviamente, la práctica de atención domiciliaria -que caracterizó a la medicina liberal- le permitía al profesional acercarse al contexto del proceso de enfermar y a las posibilidades terapéuticas de sus pacientes. En ciertas situaciones, como cuando el médico vivía en el mismo territorio que sus pacientes y, además -como era frecuente entonces- instalaba su consultorio privado en la parte delantera de su vivienda, también se generaban condiciones que favorecían una mayor familiaridad del profesional con el contexto del proceso de enfermar y con las posibilidades terapéuticas de sus pacientes.

Sin embargo, hacia fines del siglo XX, esa información complementaria comienza a ser considerada prescindible por los profesionales. Efectivamente, por un lado, la tecnología amplió la capacidad de respuesta frente a los procesos de enfermar y, con ello, el éxito técnico de la intervención. Pero esto, sin embargo, no anuló la importancia de alcanzar también un éxito práctico. Después de todo, las dificultades -de naturaleza socioeconómica o de índole más personal- para acompañar a pacientes con enfermedades crónicas, especialmente en términos de adherencia a los tratamientos, fueron siendo identificadas y reconocidas con la transición demográfica y epidemiológica de la segunda mitad del siglo XX. No obstante, las nuevas formas del mercado de trabajo y de relación con las personas asistidas, sumadas a esa ampliación del éxito técnico, forjaron en el imaginario profesional una cierta superación de la necesidad de contar con información contextual sobre el proceso de enfermar y de cuidar, respaldando el tecnicismo de la práctica que se estaba configurando.

Y como impacto práctico, en el encuentro clínico propio de la consulta en la medicina tecnológica, se produce un acortamiento del diálogo con el paciente. En el contacto directo, la conversación pasa a dividirse entre aquella considerada útil y aquella que ahora se considera inútil.

Pero es necesario considerar que esa presencia del paciente siempre se dio en el interior de una relación jerarquizada en favor del médico. La jerarquía se invertía parcialmente, de forma compensatoria para una mayor presencia del paciente, por la preocupación del médico con el acierto asistencial, dentro de un pragmatismo orientado a resolver problemas que marcó el ingreso de la medicina en la modernidad, a saber: alcanzar soluciones eminentemente prácticas formaba parte de la profesión, incluso debido a los conocimientos científicos y recursos tecnológicos entonces más limitados. Después de todo, la medicina entra en la modernidad con su lema de ser *la ciencia y el arte de curar*^8^^,^^29^^,^^30^.

Antes de que se vea en esta marca histórica una justificación para ciertos negacionismos actuales de las ciencias, vale señalar que esa sabiduría práctica -que recurre a las experiencias previas vividas por los médicos o incluso apela a un empirismo del momento (valorando lo contingente del caso clínico individual)- está bien delimitada en su articulación con el componente científico de la práctica médica. No se trata de una sustitución libre de la acción basada en la ciencia por otra basada en la experiencia empírica. Se trata, desde siempre, de una posibilidad de actuación fundamentada en la experiencia clínica del profesional, que podía darse ante la ausencia de referencias científicas o como adaptación de estas a las particularidades del caso. El recurso al empirismo, por cierto, ha sido considerado, también desde siempre, como transitorio, según la expresión del pensamiento médico sobre la profesión: *si algo no se sabe científicamente, es que aún no se sabe*. El propio lema refuerza ese valor: curar es, en primer lugar, ciencia, que puede complementarse con un uso mesurado del arte. Los procesos históricos tendieron a desplazar ese componente del arte fuera del ideal de las prácticas.

En este sentido, cabe destacar la importancia del pragmatismo de la medicina, el cual no puede reducirse a un éxito únicamente de naturaleza técnico-científica. El cuidado requiere sin duda ese éxito, pero debe articularse con un éxito práctico, el cual exige considerar, más allá de la patología, todo el contexto del proceso de enfermar y de las posibilidades concretas de cuidado; y, en esto, quien más puede decir y hacer al respecto es el propio paciente.

Así, incluso en la medicina liberal -que creó los vínculos de confianza- la complementación de información y el llamado a la participación en la consulta siempre estuvieron delimitados por la presencialidad que los médicos consideraban necesaria. Si bien esto representó cierta apertura a la voz del paciente, no era exactamente una interacción más libre o más democrática en el juego de las decisiones asistenciales, sino al menos la presencia, lo que también fue rescatado y valorado positivamente por los propios pacientes. Después de todo, una suerte de nostalgia de aquella época es expresada tanto por médicos como por pacientes, aunque desde argumentos distintos entre sí.

## EL DERRUMBE DE LO CONSTRUIDO: LA RELACIÓN ENTRE EL MÉDICO Y EL SABER

“*Hoy en día, algo que cambió mucho es el acceso a la información* [...] *Esto está generando una situación curiosa, que es el paciente que llega con información* [...] *llega ya con su carpetita* [...] *Y la paciente discutía conmigo de igual a igual -cambió la relación. Pero esa paciente va a ser tratada por mí como paciente, porque, a pesar de tener mucha información, no tiene la vivencia, la práctica* [...] *No saben para qué sirve, no saben qué es, no saben si funciona, pero ya está en los medios, ya está en el conocimiento. ¿Cómo moldeo mi postura frente a eso? ¿Qué digo? ¿Cómo me posiciono, como médico, con respecto a eso?* […] *Lo que me parece negativo es no pensar. Si la tecnología favorece eso, entonces, ese es un aspecto negativo de la tecnología, pero no estoy en contra de la medicina, no practico una medicina no tecnológica* [...] *Incluso creo que soy capaz de actuar, en mayor o menor grado, basándome en mi experiencia, pero no creo, a priori, que sea algo negativo. Y si hay aspectos negativos, también hay todo un lado positivo, que debe equilibrarse* [...] *Hoy en día soy una persona mucho más preocupada por el paciente. Cuando me gradué, estaba mucho más preocupado por el conocimiento que por el paciente en sí. Es decir -es medio grave decir esto- el paciente era un instrumento*. (Dr. Danilo, neumonólogo, entrevistado en 1997)^8^

“*La medicina hoy se basa mucho más en tecnología avanzada que en el conocimiento médico. Hoy, la ingeniería electrotécnica ha traído avances formidables para el diagnóstico, y por eso los médicos tienen que usar menos el cerebro y los clientes gastan más dinero para costear esos exámenes, llamados diagnóstico por imágenes, que para el médico son la salvación, porque las imágenes terminan haciendo el diagnóstico que el cerebro del médico no haría* [...] *Entonces, el avance tecnológico, más que el avance intelectual de los médicos, promovió el avance de la medicina. Curioso que no fue necesario usar mucho el cerebro, porque la tecnología incluso prescindió de esa cualidad. Porque acortó mucho el razonamiento médico en función de lo que ve directamente en la imagen. Eso fue lo que modificó drásticamente la relación médico-paciente. Se modificó debido al avance tecnológico, porque al contar con una máquina, un dispositivo que te da un diagnóstico seguro, rápidamente tenés el diagnóstico en la mano y rápidamente podés tomar la decisión necesaria para atender ese caso*”. (Dr. Luiz, médico generalista, entrevistado en 2009)^31^

Al considerar el plano de la relación del médico con su saber, la pérdida en las interacciones trae otros importantes impactos, tanto materiales como simbólicos. Destaco en particular la crisis en la autoridad personal del médico y el abandono del carácter reflexivo del juicio clínico. Es decir, se trata de una pérdida de la disposición ética y de la competencia práctica del médico para reflexionar sobre su propia acción: reflexionar sobre la aplicación del conocimiento científico y sobre el uso de las tecnologías materiales ante cada caso, y también reflexionar sobre cada contexto de enfermedad y de atención asistencial. Los fragmentos anteriores, presentados como epígrafe de este apartado, dan testimonio de estas situaciones, y cabe señalar el hecho de que entre el doctor Danilo y el doctor Luiz -cuyas entrevistas se realizaron respectivamente en 1997 y en 2009- median doce años de acelerado desarrollo tecnológico.

Podríamos decir que, después de la década de 1990, el médico abdica progresivamente de ejercer esa reflexividad, en favor de una aplicación directa, sin mediaciones y más mecánica, de protocolos de intervención previamente establecidos, ya sean diagnósticos o terapéuticos. Al universalizarse, tales protocolos son propuestas de intervención que se abstraen de abordar lo particular y concreto de cada caso individual y lo contingente del momento en que ocurre cada consulta clínica, ya sea para comprender el proceso de enfermar como para considerar las posibilidades de atención.

Queda claro, así, que hacia fines del siglo XX la acción médica ya se orientaba principalmente hacia la obtención del éxito técnico, quedando el éxito práctico con escasas posibilidades de estar presente como cuestión e incluso de ser revalorizado en relación con el pragmatismo de la medicina liberal.

Volvamos un poco a las raíces de estos procesos de derrumbe de la autonomía, considerando la década de 1960 como el inicio de dichos procesos y la de 1990 como un hito histórico de ruptura respecto del modo más reflexivo de operar la clínica, siempre manteniendo la base empírica ya mencionada de las narrativas de médicos sobre sus trayectorias laborales, en los estudios ya citados. Es importante recordar que las narrativas fueron construidas a partir de dos grupos de médicos entrevistados: aquellos que se graduaron entre 1930 y 1960 y que produjeron sus narraciones hacia fines de los años 1980; y aquellos que se graduaron entre 1980 y 1985 y que brindaron sus relatos hacia fines de los años 1990. Así, el impacto de los cambios fue distinto entre ambos grupos: el primero narró la aparición de las empresas en la asistencia médica, el asalariamiento, la especialización y la pérdida de importancia del consultorio. El segundo grupo, en cambio, destacó la reconfiguración del consultorio como empresa y, en ello, una reactivación de la importancia de ese espacio asistencial, narrando también la gran presencia de la tecnología material y su transformación de medio en la actividad asistencial a fin en sí mismo, cuyo acceso pasa a significar lo mejor de la atención médica. Pero también relataron el surgimiento del “médico de cartilla” o de las listas de médicos de las empresas de salud o de los seguros médicos, el anonimato al que esto los conduce frente a los pacientes y, por último, las nuevas posiciones que asumen en la relación con pacientes que también ocupan nuevas posiciones, ante la irrupción del mundo digital en la atención médica.

Así, todos estos grandes cambios se inician con el progresivo empresariamiento de los servicios y del asalariamiento de los profesionales, es decir, con el arreglo tecnológico elegido por los propios médicos para configurar la organización social de la asistencia. En Brasil, esto ocurre, primero, en el sector público de atención médica, vinculado a la seguridad social, expandiéndose posteriormente al sector privado.

El asalariamiento fue la principal cuestión de época para los profesionales entre los años 1930 y 1960, lo cual aparece en las narrativas y fue confirmado por el análisis documental del periódico *Gazeta Médica*, de circulación restringida a los propios médicos.

Pero, al valorar una autonomía que permitiera cierta libertad para cada médico individual -aunque se tratara de una libertad regida por contextos sociohistóricos bien delimitados- los profesionales, a lo largo del período, terminaron por reconocer la pérdida importante de autonomía en el plano mercantil y en la organización de sus jornadas de trabajo. Estas pérdidas implicaron también una progresiva desigualdad en las condiciones de ejercicio profesional y en las posiciones corporativas dentro del colectivo médico, señalando un movimiento de desigualdad laboral y financiera entre ellos.

Esa desigualdad al interior del cuerpo profesional no era bien reconocida entre los médicos, y creo que aún hoy no lo es. Sin embargo, en sus relatos, reconocían pérdidas importantes, como la del control sobre sus pacientes, el control sobre su jornada de trabajo, el control sobre las condiciones de remuneración y sobre los equipamientos, incluso cuando estos eran todavía de menor complejidad y, ciertamente, de menor costo.

Así, como consecuencia de los desiguales contextos empresariales, los médicos comienzan a experimentar la institucionalización de una creciente desigualdad en la prestación de servicios y en las jerarquías corporativas profesionales, lo cual se acentúa fuertemente en la segunda mitad del siglo XX. No obstante, curiosamente, se inicia un movimiento de renacimiento de la práctica en consultorio, pero de un consultorio transformado en empresa, como ya se mencionó, por sus conexiones con los engranajes socioeconómicos de producción de atención médica, como los de los planes o seguros de salud.

Por ello, esas narrativas hablan de la experiencia de la medicina liberal y de la experiencia de su transformación. Y en esta transformación, más para algunos médicos que para otros, las pérdidas tendrán impactos graves y diversos en sus cotidianos de trabajo. Se institucionalizan relaciones médico-paciente desiguales, con un aumento del carácter rutinario del trabajo y con inserciones clínicas de bajo valor, en situaciones asistenciales percibidas como poco calificadas por los propios médicos.

Inmersos en todos esos cambios, por otro lado, los médicos narran cómo acogieron lo que comprenden como una “evolución natural” de sus prácticas, a saber: la especialización del ejercicio profesional y la incorporación cada vez mayor y más acelerada de nuevas tecnologías materiales, nuevos equipamientos. Se vuelve claro, entonces, hasta qué punto la tecnología inmaterial se vio representada en el pensamiento de los médicos sobre su trabajo a través de la tecnología material, al mismo tiempo que, apropiándose progresivamente de los equipos y medicamentos como recursos tecnológicos, los entrevistados se presentan como médicos que, de hecho, se actualizaron tecnológicamente, a tal punto que ya podemos considerar la década de 1980 como el período de consolidación de la medicina tecnológica. Pero, al mismo tiempo, sus relatos señalan algunos límites a esa actualización. Destacan precauciones importantes en el uso de los nuevos medicamentos y mencionan indicaciones más moderadas de exámenes diagnósticos, prefiriendo la anamnesis extensa, con un mayor tiempo de conversación con el paciente y privilegiando sus propios logros asistenciales. Por eso, estos médicos se representan a sí mismos como un grupo generacional más identificado con ciertas resistencias que con las transformaciones, aceptando mucho más aquellas que ocurrieron en su mercado de trabajo que las que se relacionan con el segmento técnico-científico.

Y lo hacen así, quizás por sospechar lo que está por venir a partir de los años 1990: la gran tensión sobre la autonomía en el juicio clínico, la autonomía de y en la consulta médica, que afecta directamente a la decisión asistencial.

Con el pasaje de una medicina liberal a una medicina tecno-empresarial, cambia el carácter de un servicio que se había configurado con cierto desprendimiento mercantil -porque buscó identificarse con una “profesión noble” y no con un “negocio”- a una medicina declaradamente orientada al negocio^8^, cada vez más inserta en el polo más radical del mercado, el capitalismo rentista. También cambia la relación interpersonal del médico con sus pacientes, que se transforma en un contrato jurídico con los seguros de salud o planes médicos; así como se pasa de una práctica centrada en la persona del médico a una asistencia en la que participan afiliados anónimos y médicos anónimos pertenecientes a las cartillas de los seguros de salud.

Frente a todo esto, los médicos fueron reconociendo, poco a poco, que si se producía el pasaje del médico que salva al medicamento que salva -como efectivamente vieron suceder en sus trayectorias laborales- también se producía el pasaje de la confianza en *mi* médico, a la confianza en la tecnología del hospital.

El punto crítico de la profesión pasa, entonces, a situarse en el ámbito de los vínculos interactivos del encuentro clínico, en los que también la presencia del propio médico, como sujeto actuante de su práctica, ha perdido la centralidad que tenía anteriormente. Su presencia parece ser, del mismo modo, una instancia de paso, un acceso a las tecnologías que salvan. Y este proceso también ha generado una crisis identitaria de los médicos con respecto a la medicina, debido a la crisis que ha recaído también sobre su autoridad, como representante del polo científico en la práctica asistencial.

Como configuración general de la práctica médica, interpreté estos procesos como una ruptura histórica con las referencias de la medicina liberal; referencias que podrían haber orientado los desempeños profesionales. Esto significa, siguiendo el pensamiento de Arendt^32^, una situación de “crisis” en la cual, frente a los problemas vividos en el presente, el pasado no puede ofrecer referencias para resolverlos. Esto sitúa al médico de otra manera frente al tradicional poder superior que había conquistado en sus relaciones técnico-asistenciales.

Pero, por otro lado, la concepción ideal de la “buena práctica” aún retiene ciertos elementos del modelo liberal, en la figura del médico siempre disponible y consejero de la familia. En una formulación claramente ideológica, este ideal se reitera como modelo a ser perseguido por todos los médicos. Y, siguiendo a Bourdieu^33^, ese ideal se mantiene, para la población en general, como la cultura legítima del campo de la salud.

Sin embargo, en el cotidiano del trabajo, hay una distancia entre el ideal y lo efectivamente practicado, y de esa distancia se deriva el profundo extrañamiento de los médicos frente a lo que viven en sus servicios^16^^,^^17^^,^^31^. Ese extrañamiento se extiende hasta nuestros días.

La manera en que los propios médicos reaccionan y lidian con ese extrañamiento termina por inscribir nuevas y relevantes problemáticas. Muy discutidas en Brasil, estas se agrupan en torno a los ejes de la “deshumanización” y de la “violencia” en los servicios de salud, ya sea ejercida por los profesionales o por las instituciones.

Una vez más, cabe considerar el pensamiento arendtiano, que distingue entre violencia y poder. Donde hay uno, no habrá el otro, dirá la pensadora^34^. Es decir, la violencia no sería un exceso de poder, como frecuentemente se la considera en textos del campo de la salud y también en la salud colectiva^28^. Según Arendt, el poder es el reconocimiento de una autoridad legítima, y su uso instrumental en acciones intencionadas por determinados intereses no produce interacción ni acción ético-comunicativa, siguiendo a Habermas^35^. Al contrario, produce una imposición al Otro, de afirmaciones y decisiones unilaterales. Y esta es una forma autoritaria de relación, transformada de una interacción entre sujetos en una relación de mando-obediencia, cuyo nombre es “violencia” y no “poder”.

En este sentido, el pasaje de una actitud de extrañamiento por parte de los profesionales hacia una práctica de convencimiento de los pacientes -acerca de que el mejor juicio y la mejor decisión son los que formulan los médicos- se presenta como una disposición autoritaria. Y ese carácter se refuerza cuando los médicos señalan que, en ese convencimiento, sería posible e incluso deseable, en nombre de la ciencia, apelar a ciertas coerciones o, como dicen, a “pequeñas presiones” en favor de un bien que consideran superior. Esto ya constituye una cuestión relevante en relación con la legitimación de la autoridad, más aún si se considera la perspectiva de los derechos humanos en este tipo de relación. En otros términos, ¿sería posible hablar de coerciones que serían pequeñas tratándose de contextos asistenciales?

Y aquí debo añadir otra cuestión: ¿ese procedimiento de imposición sería restaurador de la confianza? ¿Restauraría el reconocimiento de la autonomía y de la legítima mayor autoridad del médico? ¿Y sería posible pensar en una restauración aislada de la autonomía técnico-científica frente a los nuevos contextos de producción asistencial de mercado y de gestión de esa producción en el cotidiano del trabajo? ¿Dónde “anclar” ahora los vínculos de confianza? ¿Y pensando en términos tecnológicos, cómo recuperar la autoridad del sujeto en el proceso de creación y definición del uso de la tecnología?

Debo considerar que, a mediados de la década de 1990, en una investigación publicada en 1996 sobre el mercado de trabajo de los médicos en Brasil^36^, ante la pregunta sobre cómo veían el futuro de la profesión, los médicos entrevistados respondían, en lo que podría sintetizarse como: *“A la medicina le va bien, a los médicos, no tanto”*.

Se rompía, entonces, un vínculo importante en la relación de los médicos con la medicina. Pero lo sorprendente es que, hoy en día, constataríamos que, en definitiva, *la medicina ya no va tan bien*. Desde entonces, el derrumbe de lo que fue construido se ha ido expandiendo, y la crisis de confianza se ha extendido, alcanzando incluso a las ciencias, como lo evidencia el negacionismo de la actualidad. Un negacionismo que, en el contexto pandémico de los años 2020-2022, manifestaba tanto una crisis de confianza de carácter comunicativo entre los especialistas y la población -especialmente en lo que respecta a las incertidumbres en la medicina- como una crisis de confianza entre los médicos y el saber médico, en la que parte de ellos invocaba la autonomía del juicio clínico que venían perdiendo desde mucho antes de la pandemia.

En este momento, me gustaría entrelazar el enfoque histórico que vengo desarrollando con la cuestión, muy actual, de la autonomía de los médicos en medio del negacionismo de las ciencias. Quisiera intentar esta articulación no solo por el contexto pandémico que todos hemos atravesado, sino también, por supuesto, debido a la importancia de la memoria en relación con el trágico escenario brasileño.

Quiero hacer mención a una publicación en una revista brasileña de periodismo investigativo y con una línea bastante crítica. Se trata de la revista *Piauí*, en la que Tatiana Roque^37^ publicó en 2021 el artículo titulado “*A queda dos experts*” (La caída de los expertos). El artículo resulta provocador ya desde su título, sobre todo si recordamos la crítica que Arendt dirige a la modernidad, respecto de la constitución de la sociedad de los especialistas, en la que los expertos gobiernan lo social, ocupando el lugar que corresponde a la acción política. Pero la acción política es producto de la interacción en y del espacio público, y no la opinión unilateral de especialistas dirigida al público. ¿Y cuál sería, en esta dirección, la competencia de los médicos? Después de todo, estos especialistas buscaron precisamente no involucrarse con la acción política, construyendo un mundo aparte de lo social y neutralizando sus acciones. ¿Cómo comprender, entonces, una acción política tan despolitizada? ¿Cómo sostener una acción política fundada en una duda negacionista acerca de la propia neutralidad ético-política que los médicos han pretendido históricamente al construir el carácter estrictamente científico-tecnológico de su intervención asistencial?

Tatiana Roque es profesora del Instituto de Matemática de la Universidade Federal do Río de Janeiro, con formación en Matemática, Historia de las Ciencias y Filosofía. Viene trabajando sobre la relación de las mujeres con la ciencia desde una perspectiva histórica. Escribió para la misma revista otro artículo, publicado en 2020, titulado “*O negacionismo no poder*” (El negacionismo en el poder)^38^. En ambos textos, la autora aborda una crisis de confianza: en la ciencia, en relación con el negacionismo y, en los *expertos*, en relación con la pérdida del poder de los especialistas. Quiero subrayar que elijo este camino de abordaje justamente porque estos artículos no constituyen una comunicación entre pares, es decir, entre científicos o entre tecnólogos, agentes que recurren a la ciencia en sus prácticas cotidianas. Esta comunicación tiene que ver con una científica que quiere dialogar con lectores que no forman un colectivo estrictamente compuesto por científicos, aunque el tipo de revista ya delimite al público lector, en términos culturales y de estratificación socioeconómica.

En su texto, Tatiana desarrolla la noción del debilitamiento de la credibilidad de los *expertos*, a quienes define como investigadores que median entre la ciencia y la política, y que se ven muy comprometidos y generan conflictos cuando tratan lo político a través de los dispositivos científicos de comunicación/interacción con los que están habituados como agentes de la ciencia. Las consideraciones de Tatiana me sugieren que los especialistas actúan desde disposiciones propias del campo científico, pero aplicadas al terreno de la política, y se equivocan al equiparar un dominio con el otro: el de las ciencias y el de la política.

Por ejemplo, la autora señala que, en la reciente pandemia, la Organización Mundial de la Salud se vio comprometida en declaraciones contradictorias, aun cuando estuvieran basadas en evidencia científica. Ese tipo de declaraciones representa un gran problema en la comunicación pública que pretende orientar políticas de Estado, pero no lo es en la comunicación científica. Después de todo, para la comunidad científica existe el entendimiento del conocimiento como una verdad temporal, limitada por las opciones metodológicas. Así, la diversidad de resultados, que puede provocar conflictos entre investigadores, no hace más que estimular nuevos estudios con nuevas elecciones de diseño y método.

Tal situación remite, particularmente en el campo de la salud, a las incertidumbres siempre presentes en la práctica médica, y que son abordadas de modo muy diverso por los médicos o, en ocasiones, incluso desconsideradas en sus comunicaciones con los pacientes. Las incertidumbres, que anteriormente podían ser manejadas a través de la acción cautelosa y criteriosa de cada médico en cada caso, ya no encuentran el mismo respaldo personal en el tecnicismo de la medicina tecnológica. Y el optimismo tecnológico muchas veces contrasta con complicaciones inesperadas -aunque posibles- en el espectro de las incertidumbres, generando extrañamiento en los pacientes respecto de la calidad de la atención recibida.

Tatiana^37^ también propone la idea de que la crisis actual de los *expertos* y de sus instituciones está relacionada con el éxito que obtuvieron hasta ahora, señalando que el respaldo científico funcionó durante mucho tiempo, en tanto fue capaz de demostrar el aumento de la calidad de vida a partir de sus tecnologías. Agrega, además, que actualmente ha crecido la percepción de los riesgos inherentes a dichas tecnologías.

Creo que podemos ponderar, siguiendo esta línea argumentativa, que la propia tecnología ha acortado el tiempo de latencia entre el uso de ciertos dispositivos y los impactos -positivos o negativos- derivados de su utilización, permitiéndonos poner en discusión la utilidad misma de tal o cual tecnología. Y aquí resulta central el esclarecimiento de las incertidumbres en el terreno de la ciencia y de la tecnología en medicina.

Tatiana^37^ plantea además dos cuestiones importantes: una, respecto de que no debemos transformar la opinión de los *expertos*, basada en las ciencias, en una decisión política para la sociedad, pues esa transformación -afirma- acentúa la crisis de confianza; la otra, se refiere justamente a cómo restaurar la confianza en las ciencias y sus instituciones, a lo que agrego: también la confianza en la política democrática y en sus instituciones.

Sobre la primera cuestión -la transformación de la opinión de los *expertos* en decisión de política pública-, señala que el ritmo creciente en la producción de vacunas, por ejemplo, muestra cuánto puede beneficiarnos la ciencia. Pero el intento de convertir esa capacidad en moneda de cambio para obtener visibilidad mediática -ganando más espacio como espectáculo que desde la eficacia en la prevención de muertes y enfermedades- o de usar esa capacidad científica como instrumento de lucro financiero oportunista y antiético, solo refuerza el desastre material producto de tales oportunismos y del propio negacionismo.

A mi entender, aquí cabe reforzar otra cuestión: hay quienes asocian todo ese instrumentalismo financiero o mediático a un exceso de “politización” de las ciencias y sus tecnologías. Pienso que, por el contrario, esa actitud y las intervenciones que de ella se derivan despolitizan la acción, transformándola en instrumento de intereses privados y no en un espacio de elección pública, lo que podríamos comprender mejor como un *instrumentalismo de la acción*, la cual pierde su carácter ético-político para asumir el carácter de una relación de mando-obediencia, y ya no de una interacción ético-política, engendrando así una “política despolitizada”.

Además, este tipo de acción orientada al interés privado no se produce como una elección en torno a un “común”, tal como exige la esfera pública. Evitar ese instrumentalismo oportunista no implica neutralizar las ciencias ni el uso de las tecnologías. En tanto práctica social, la científica está siempre atravesada por lo político, ya que forma parte de las elecciones humanas que configuran los contextos sociohistóricos. Y, en ese sentido, corresponde respetar y entrar en contacto con esa politización y, en el marco de una decisión colectiva en la esfera pública, preguntarnos ¿qué queremos conocer científicamente?, ¿qué tecnologías deseamos tener?, ¿para producir qué tipo de utilidad?. Vale aquí recordar lo que dice Arendt a propósito de las tecnologías y sus utilidades para los seres humanos: que las ciencias sirven a los humanos y que las tecnologías son productos dotados de utilidades, eso no se discute, pero que la decisión sobre cuál utilidad queremos, en tal o cual momento y contexto, es política, no científica ni tecnológica^3^.

Una última consideración debe hacerse, en particular, sobre cómo restaurar la confianza en las ciencias y en sus instituciones. Dirá Tatiana Roque^37^: recuperar la confianza significa mejorar la reputación de las instituciones democráticas, entre ellas, la institución científica. Y, en esto, destaca dos caminos: el primero es establecer una clara distinción entre lo que es divulgación científica y lo que es asesoramiento para políticas públicas; y, el segundo, se relaciona a esta función de asesoramiento, la cual debería evitarse que sea de carácter personal, priorizando los consensos colectivos que amplían el debate. Más importante que la persuasión ejercida por los especialistas es la posibilidad de que exista debate público y democrático. Así, restaurar la confianza es un proceso que implica dar voz, dar lugar a la participación en las decisiones.

En el campo de la salud, contamos con conferencias municipales, consejos comunitarios, e incluso debates globales, como se observa en las discusiones sobre el clima y la preservación del planeta. Se trata, ahora, de implementar de manera efectiva esos foros más amplios y de mejorar nuestra capacidad para el debate. Y, además de ello, es necesario promover también, como espacio de intercambio en participación dialógica, el momento de toma de decisiones en torno a los cuidados asistenciales. Es decir, que los profesionales abran la posibilidad de una decisión compartida con el paciente en lo que respecta a su plan de atención, y compartida también con los diversos profesionales que integran el trabajo en equipo en los servicios de salud. Con ello se promoverían espacios de reconocimiento de los saberes de los pacientes y de los profesionales de salud de las distintas áreas. Se espera que, con estas aperturas, sea posible hacer frente al derecho a la salud sin reducirlo al mero consumo de servicios regido por la intensa mercantilización y financiarización del sector salud.

Y esto, sin duda, requiere una disposición para interactuar con la diversidad de opiniones, interactuar con la pluralidad de sujetos que somos. Y si los recursos tecnológicos pueden facilitar ese empeño, esa será la utilidad de la tecnología que queremos. En cuanto a otras utilidades... aún queda debatir y decidir.
